# Targeted agents for patients with advanced/metastatic pancreatic cancer: A protocol for systematic review and network meta-analysis

**DOI:** 10.1097/MD.0000000000010115

**Published:** 2018-03-30

**Authors:** Baoshan Di, Bei Pan, Long Ge, Jichun Ma, Yiting Wu, Tiankang Guo

**Affiliations:** a Department of Emergency, Gansu Provincial Hospital West Campus; b Department of Social Medicine and Health Management, School of Public Health; c The First Clinical Medical College, Lanzhou University; d Evidence-based Medicine Center, School of Basic Medical Sciences, Lanzhou University; e School of Clinical Medicine, Gansu University of Traditional Chinese Medicine, Lanzhou, China.

**Keywords:** Bayesian, network meta-analysis, pancreatic cancer, protocol, targeted agents

## Abstract

**Background:**

Pancreatic cancer (PC) is a devastating malignant tumor. Although surgical resection may offer a good prognosis and prolong survival, approximately 80% patients with PC are always diagnosed as unresectable tumor. National Comprehensive Cancer Network's (NCCN) recommended gemcitabine-based chemotherapy as efficient treatment. While, according to recent studies, targeted agents might be a better available option for advanced or metastatic pancreatic cancer patients. The aim of this systematic review and network meta-analysis will be to examine the differences of different targeted interventions for advanced/metastatic PC patients.

**Methods:**

We will conduct this systematic review and network meta-analysis using Bayesian method and according to Preferred Reporting Items for Systematic review and Meta-Analysis Protocols (PRISMA-P) statement. To identify relevant studies, 6 electronic databases including PubMed, EMBASE, the Cochrane Central Register of Controlled Trials (CENTRAL), Web of science, CNKI (Chinese National Knowledge Infrastructure), and CBM (Chinese Biological Medical Database) will be searched. The risk of bias in included randomized controlled trials (RCTs) will be assessed using the Cochrane Handbook version 5.1.0. And we will use GRADE approach to assess the quality of evidence from network meta-analysis. Data will be analyzed using R 3.4.1 software.

**Results and conclusion:**

To the best of our knowledge, this systematic review and network meta-analysis will firstly use both direct and indirect evidence to compare the differences of different targeted agents and targeted agents plus chemotherapy for advanced/metastatic pancreatic cancer patients. This is a protocol of systematic review and meta-analysis, so the ethical approval and patient consent are not required. We will disseminate the results of this review by submitting to a peer-reviewed journal.

## 1 Introduction

Pancreatic cancer (PC), which is derived from the glandular tissue of the pancreas, is a devastating malignant tumor and characterize with high mortality and poor prognosis.^[1]^ And the incidence of PC is increasingly rising. PC is the fourth major cause of cancer-related death in all population worldwide; it causes about 338,000 new cases each year.^[2]^ The 5-year survival rate of PC patients with R0 pancreatic surgery is 6%, and the median overall survival time is 4 to 6 months in patients with metastatic disease.^[3]^ Although surgical resection may offer a good prognosis and prolong survival, approximately 80% patients with PC are always diagnosed as unresectable tumor (locally advanced and/or metastatic), because the symptoms of PC generally occur late, and it will lead to the extremely poor prognosis for advanced PC.

Chemotherapy is considered as a major treatment for unresectable PC, and according to the National Comprehensive Cancer Network's (NCCN) recommendation, gemcitabine-based chemotherapy is a standard backbone for advanced/metastatic PC.^[4,5]^ However, recent studies showed that better therapeutic options are available for advanced/metastatic PC, such as target therapies.^[6,7]^ Both erlotinib (Tarceva), everolimus (Afinitor) and sunitinib (Sutent) were global approved for management and treatment of advanced PC and prolonged progression-free survival (PFS) for PC patients. Sunitinib malate is an oral small-molecule tyrosine kinase inhibitor.^[8–10]^ Erlotinib, an orally bioavailable inhibitor of EGFR, has been clinically approved for unresectable pancreatic cancer in combination with gemcitabine.^[11]^ Everolimus as the mammalian target of rapamycin inhibitor is demonstrated that can significantly prolong PFS in patients with advanced PC.^[12]^

Wang's meta-analysis focused on gemcitabine plus erlotinib for locally advanced/metastatic PC. Vinik's systematic review and meta-analysis focused on sunitinib for the quality of life of advanced PC patients.^[13]^ And everolimus may prolong PFS and down-regulates excess production of 2 gastrointestinal hormones in patients with PC.^[14]^ However, there was no meta-analysis to compare the efficacy of different targeted agents for PC patients. Thus, we cannot determine which targeted agent is superior to other treatment using randomized controlled trial or pairwise comparison meta-analysis.

In the network meta-analysis, the available information from pairwise comparisons of treatment A and treatment B is combined with indirect comparisons C either a third intervention or a control condition to estimate the relative effectiveness among all interventions and rank ordering of the interventions even if head-to-head comparisons are lacking.^[15]^

Present systematic review and network meta-analysis will evaluate the relative efficacy of different targeted agents combined with chemotherapy for advanced/metastatic PC in the improvement of overall survival, progression-free survival, and adverse events using Bayesian network meta-analysis.

## 2 Methods

This protocol will be reported according to preferred reporting items for systematic review and meta-analysis protocols (PRISMA-P).^[16]^ The study protocol has been registered on the international prospective register of systematic review (PROSPERO) (CRD42017076728).

### 2.1 Eligibility criteria

Studies will be included in this systematic review and network meta-analysis if meet the following eligibility criteria: randomized controlled trials (RCTs); patients from all countries and the age is more than 18; patients that are diagnosed with advanced/metastatic PC; studies focused on the differences between different targeted agents and/ or chemotherapy; the outcome is progression-free survival, overall survival, and adverse events; there will be no limitations on year of publication, publication status, and language of publication.

### 2.2 Literature search and study selection

To identify relevant studies, the following 6 electronic databases will be searched: PubMed, EMBASE, the Cochrane Central Register of Controlled Trials (CENTRAL), Web of science, CNKI (Chinese National Knowledge Infrastructure), and CBM (Chinese Biological Medical Database). Also we will manually search the bibliographies of included articles and relevant systematic reviews and meta-analysis to identify other additional studies.

The following search terms will be used: pancreatic neoplasm, pancreatic cancer, cetuximab, erlotinib, tarceva, everolimus, afinitor, sunitinib, sutent, bevacizumab, trastuzumab, trametinib, ganitumab, and ruxolitinib.

Two authors will independently screen the title and abstract of retrieved studies. Moreover, the potentially eligible studies will be assessed by retrieving the full texts. In addition, a third reviewer will be requested in case of disagreement. We will use a flow diagram to illustrate the study selection process according to PRISMA guidelines.^[17]^

### 2.3 Data collection

#### 2.3.1 Data management

We will first perform a pilot test between 2 reviewers to ensure high inter-rater reliability. Then the management of literature search records will be conducted in ENDNOTE X7.

#### 2.3.2 Data extraction

A standard data extraction form will be created using Microsoft Excel 2013 (Microsoft Corp, Redmond, WA, www.microsoft.com) to collect data of interest, which including general characteristics of included trials (e.g., name of first author, year of publication, whether single-center or multicenter, country of study, recruitment time frame, follow-up length, total sample size, inclusion and exclusion criteria), details of participants (e.g., gender, age, tumor stage, tumor size, lymph node status), details of interventions (e.g., regimens of interventions, dosage), and outcomes.

#### 2.3.3 Quality assessment

The quality of a body of evidence will be assessed by paired reviewers with the Grades of Recommendation, Assessment, Development and Evaluation (GRADE) approach.^[18]^ Any conflicts will be resolved by consulting an independent adjudicator. Direct evidence from RCTs will start at high quality and can be rated down based on risk of bias, indirectness, inconsistency, and publication bias. The rating of indirect estimates will start at the lowest rating of the 2 direct estimates that contribute as the first-order loops to the indirect estimate but will be rated down further for intransitivity. If direct and indirect estimates contribute similar power to the network estimate, then we will use the higher rating as the rating of network meta-analysis. The network meta-analysis will be further rated down if they are inconsistency and imprecision.

#### 2.3.4 Risk of bias of included studies

The risk of bias of included studies will be estimated using the Cochrane Handbook version 5.1.0^[19]^ tool, which includes 7 specific domains: sequence generation (selection bias), allocation concealment (selection bias), blinding of participants and personnel (performance bias and detection bias), incomplete outcome data (attrition bias), selective outcome reporting (reporting bias), and other bias. Based on criteria for judging the risk of bias,^[19]^ we will classify methodological quality as low risk of bias “+,” high risk of bias “−,” or unclear risk of bias “?” Two independent reviewers will complete the assessment of risk of bias. The conflicts will be resolved by a third reviewer.

#### 2.3.5 Data synthesis

We will use Microsoft Excel 2013 to design a form summarize data of all the included studies and showing their major characteristics and some important information related to this systematic review and meta-analysis. We will use STATA version 12.0 software to combine data and conduct a pairwise meta-analysis. *I*^2^ statistic will be used to conduct heterogeneity assessment. If 0≤*I*^2^≤25%, we consider statistical heterogeneity as small; as medium if 25% < *I*^2^≤50%; as large if *I*^2^> 50%.^[20]^ Random-effect model will be used if the heterogeneity exists, otherwise, fixed-effect model analysis will be performed. We will use pooled odds ratios (ORs) with 95% confidence interval (95%CI) to show dichotomous outcomes, and use standard mean difference (SMD) or weight mean difference (WMD) with 95% CI for continuous outcomes.

We will use R-3.4.1 software and package *gemtc* version 0.9-2 to conduct a Bayesian network meta-analysis.^[21]^ We will use node splitting method to evaluate inconsistency between direct and indirect comparisons if a loop connecting 3 arms exists. The treatment ranking will be presented based on the point estimates and standard errors of the network assess.

#### 2.3.6 Subgroup and sensitivity analyses

Considered of possible significant heterogeneity or inconsistency, we will use subgroup analysis to find the possible sources. We also will estimate the sensitivity of results according to the results of risk of bias.

#### 2.3.7 Publication bias

We will use STATA version 12.0 software (Stata Corporation, College Station, TX) to draw a comparison-adjusted funnel plot to identify whether there will be a small sample effect among the networks.

## 3 Author contributions

DBS, GL, CN, and GTK planned and designed the research; GL, PB, and MJC tested the feasibility of the study; DBS and PB wrote the manuscript; all authors approved the final version of the manuscript.

**Conceptualization:** B. Di, L. Ge, T. Guo.

**Data curation:** B. Pan, J. Ma.

**Investigation:** B. Di, B. Pan, J. Ma, Y. Wu.

**Methodology:** B. Di, L. Ge.

**Supervision:** T. Guo.

**Writing – original draft:** B. Di, B. Pan.

**Writing – review & editing:** B. Di, J. Ma, L. Ge, T. Guo, Y. Wu.
